# Primary Cardiovascular Prevention in People Living With HIV: Rethinking Statin Therapy in the Post-REPRIEVE Era

**DOI:** 10.1007/s11904-026-00797-w

**Published:** 2026-09-16

**Authors:** Paolo Fusco, Francesco De Maria, Bruno Tassone, Alessandro Russo

**Affiliations:** 1 Infectious and Tropical Diseases Unit, Department of Medical and Surgical Sciences, “Magna Graecia” University of Catanzaro, Catanzaro, Italy; 2 Department of Medical and Surgical Sciences, “Magna Graecia” University, Catanzaro, Italy

**Keywords:** HIV, Cardiovascular disease, Statins, Primary prevention, Antiretroviral therapy, Drug–drug interactions

## Abstract

**Purpose of Review:**

This review examines approaches to primary cardiovascular prevention in people with HIV (PWH), focusing on the implications of the REPRIEVE trial and the 2026 ACC/AHA multisociety dyslipidemia guideline. We discuss cardiovascular risk mechanisms, limitations of conventional prediction tools, selection and monitoring of statin therapy, and the potential roles of imaging and non-statin lipid-lowering agents.

**Recent Findings:**

REPRIEVE demonstrated that pitavastatin reduced major adverse cardiovascular events in PWH aged 40–75 years receiving antiretroviral therapy and at low-to-moderate estimated cardiovascular risk. Absolute benefit varied substantially according to baseline risk. Current US guidance recommends statin therapy for primary prevention in PWH aged 40–75 years receiving stable combination antiretroviral therapy, while quantitative risk assessment remains important for communicating expected benefit and informing treatment intensity. REPRIEVE did not directly study PWH younger than 40 years or older than 75 years; however, cohort data suggest a higher HIV-attributable relative cardiovascular risk at younger ages despite low absolute event rates, whereas general-population evidence supports statin efficacy and acceptable tolerability in older adults. Conventional risk models show variable performance in PWH, and high-quality evidence supporting imaging-guided prevention or non-statin cardiovascular outcome reduction specifically in this population remains limited.

**Summary:**

Statin therapy is an evidence-based component of primary cardiovascular prevention in PWH aged 40–75 years, but guideline-level eligibility does not imply uniform absolute benefit. Outside the REPRIEVE age range, decisions should integrate the distinct balance of relative and absolute risk, general-population evidence, comorbidity, frailty, drug–drug interactions, treatment burden, and individual preferences rather than rely on age alone.

**Supplementary Information:**

The online version contains supplementary material available at https://doi.org/10.1007/s11904-026-00797-w.

## Introduction

Cardiovascular disease has emerged as a leading cause of morbidity among people living with HIV (PWH). With the widespread use of effective antiretroviral therapy (ART), HIV has evolved into a chronic condition, and non-AIDS comorbidities now account for an increasing share of the overall disease burden [1, 2].

Epidemiologic studies have consistently reported a 1.5–2-fold higher risk of myocardial infarction and other cardiovascular events in PWH compared with individuals without HIV [1–3]. This excess cardiovascular burden provides a strong rationale for improved preventive strategies, while the relative contributions of conventional, behavioral, treatment-related, and HIV-specific mechanisms are discussed in detail below.

Beyond traditional lipid-driven pathways, persistent immune activation and systemic inflammation remain central drivers of atherogenesis despite virologic suppression. These processes contribute to endothelial dysfunction, plaque formation, and thrombosis, ultimately resulting in a distinct and incompletely understood cardiovascular phenotype in PWH [4–6].

Given the higher observed incidence of cardiovascular events in PWH compared with the general population [7, 8], conventional risk models may underestimate cardiovascular risk, particularly among individuals categorized as having low or intermediate predicted risk [9]. Available validation studies suggest that underestimation may be especially relevant among younger PWH, women, and Black individuals [9]. Accordingly, preventive decisions should not rely exclusively on numerical risk estimates but should integrate conventional risk factors, relevant HIV-related risk enhancers, and individualized clinical judgment.

The REPRIEVE trial provided the first large-scale randomized evidence that statin therapy reduces major adverse cardiovascular events (MACE) among PWH aged 40–75 years receiving ART and at low-to-moderate estimated cardiovascular risk [10]. These findings have substantially expanded the evidence base for primary cardiovascular prevention in middle-aged and older PWH, while also raising important questions regarding how baseline absolute risk, HIV-related risk enhancers, treatment burden, and individual preferences should inform statin initiation in clinical practice.

## Pathogenesis of Cardiovascular Risk in HIV

Cardiovascular risk in PWH extends beyond traditional lipid-mediated mechanisms and is increasingly recognized as resulting from a complex interplay between persistent immune activation, chronic inflammation, and metabolic factors [3].

Importantly, the excess cardiovascular risk observed in PWH should not be attributed solely to HIV-specific biological mechanisms. Many HIV cohorts have historically been characterized by a disproportionate burden of conventional and behavioral cardiovascular risk factors, particularly cigarette smoking, together with hazardous alcohol use, other substance use, and adverse psychosocial and socioeconomic factors [3, 11]. In a nationally representative US comparison, 42.4% of adults with HIV receiving medical care were current smokers, compared with 20.6% of adults in the general population; among PWH, smoking was also associated with poverty, homelessness, incarceration, substance use, binge alcohol use, and depression [11]. Although multivariable analyses can account for measured covariates, differences in the intensity, duration, clustering, and ascertainment of these exposures may result in residual or unmeasured confounding. Thus, the excess cardiovascular risk reported in observational cohorts likely reflects overlapping contributions from conventional and behavioral risk factors and HIV-related mechanisms, whose relative effects cannot always be clearly disentangled.

Despite effective viral suppression, chronic immune activation persists and is driven by multiple mechanisms, including residual viral replication, microbial translocation from the gastrointestinal tract, and co-infections. This state is characterized by sustained T-cell and monocyte activation and increased production of pro-inflammatory cytokines. Elevated levels of interleukin-6, D-dimer, and high-sensitivity C-reactive protein have been consistently associated with cardiovascular events and mortality, supporting a key role of inflammation in HIV-related atherogenesis [5, 6]. In this context, biomarkers of monocyte activation, such as soluble CD14, have also been linked to adverse clinical outcomes [12].

Endothelial dysfunction represents a key intermediate step linking immune activation to vascular disease and is mediated by inflammation, oxidative stress, and immune-mediated injury [3, 4]. Imaging studies have demonstrated a higher prevalence of non-calcified and potentially vulnerable atherosclerotic plaques in PWH compared with HIV-negative individuals, suggesting a distinct and potentially more inflammatory atherosclerotic phenotype [13].

ART may further modulate cardiovascular risk through drug-specific effects. Observational studies initially identified an association between recent abacavir exposure and an increased risk of myocardial infarction [14]. This concern has been reinforced by a recent analysis of the REPRIEVE cohort including 6,114 participants receiving abacavir, tenofovir alafenamide, or tenofovir disoproxil fumarate at trial entry. In baseline-adjusted analyses, the hazard of first MACE was directionally higher with abacavir than with tenofovir alafenamide (hazard ratio [HR], 1.5; 95% CI, 0.9–2.3) or tenofovir disoproxil fumarate (HR, 1.4; 95% CI, 0.9–2.1). After censoring at the first NRTI switch and applying inverse probability-of-censoring weighting, abacavir was associated with a higher MACE hazard than tenofovir disoproxil fumarate (HR, 2.0; 95% CI, 1.2–3.4), with the largest estimate observed for myocardial infarction (HR, 3.5; 95% CI, 1.3–9.4) [15]. Although NRTI exposure was not randomized and residual confounding cannot be excluded, these findings strengthen concerns regarding the cardiovascular safety of abacavir and support careful consideration of cardiovascular risk when selecting an antiretroviral regimen.

In addition, contemporary ART regimens may influence cardiometabolic risk through weight gain and metabolic changes. In particular, integrase strand transfer inhibitor (INSTI)-based regimens, especially when combined with tenofovir alafenamide (TAF), have been associated with significant weight gain and adverse metabolic profiles in some studies, although the long-term impact on cardiovascular outcomes remains uncertain [16].

Furthermore, certain antiretroviral agents may exert drug-specific effects on cardiovascular risk. A large observational study has suggested an increased risk of cardiovascular disease with cumulative exposure to darunavir/ritonavir (DRV/r), although this association has not been consistently confirmed across studies [17]. Accordingly, current guidelines recommend caution when using DRV/r in individuals at high cardiovascular risk [18].

Overall, these mechanisms converge to produce an inflammatory and immune-mediated component of cardiovascular risk that is not adequately captured by traditional lipid measurements or risk prediction models, highlighting the need for a broader pathophysiological framework in cardiovascular risk assessment in PWH.

### Limitations of Cardiovascular Risk Prediction

Cardiovascular risk models developed for the general population, including the Pooled Cohort Equations (PCE), Framingham-based equations, and SCORE-based approaches, show variable discrimination and calibration when applied to PWH. These models do not directly incorporate potentially relevant HIV-related variables, such as prolonged exposure to viremia, low nadir CD4 + T-cell count, selected antiretroviral exposures, or persistent immune activation. Validation studies summarized by Achhra et al. identified a general tendency toward risk underestimation, particularly among younger PWH, women, Black individuals, and those classified as having low or intermediate predicted risk [9]. However, miscalibration is not uniform across all models or populations, and the HIV-specific D: A:D model has also shown only modest external performance, particularly in US-based cohorts [9]. No single risk equation can therefore be considered universally optimal for PWH; risk estimates should be interpreted as a structured starting point for clinical assessment rather than as definitive treatment thresholds.

Carotid intima–media thickness (cIMT) has been widely used as a non-invasive surrogate marker of subclinical atherosclerosis in PWH. Earlier longitudinal studies demonstrated accelerated cIMT progression and increased carotid plaque burden in PWH, supporting the concept that HIV-related mechanisms may contribute to vascular aging beyond traditional risk factors [19, 20].

However, cIMT remains a surrogate endpoint and its use in routine cardiovascular risk stratification is limited by variability in measurement protocols, lack of standardized thresholds, and weaker incremental predictive value compared with coronary imaging. Nevertheless, it represents a well-established marker of early atherosclerosis and has been widely used in cardiovascular risk research, particularly in populations where long-term outcome data are limited [21].

Coronary artery calcium (CAC) scoring can improve cardiovascular risk classification in selected individuals and has also been explored in PWH. In a cross-sectional study of 739 PWH aged > 50 years, CAC scoring reclassified cardiovascular risk in a substantial proportion of participants compared with conventional risk scores, suggesting potential value when estimated risk and subclinical atherosclerotic burden are discordant [22]. However, these findings derive from observational data and do not establish that CAC-guided preventive strategies reduce cardiovascular events. More broadly, no randomized trial has demonstrated that CAC-guided management improves hard cardiovascular outcomes specifically in PWH, and the available HIV-specific evidence remains predominantly observational [22, 23]. Moreover, CAC quantifies calcified plaque and may incompletely characterize coronary atherosclerosis in PWH, in whom non-calcified plaque is relatively common [13, 23].

CAC testing also carries potential disadvantages, including radiation exposure, incidental findings, and downstream diagnostic testing; in asymptomatic individuals, abnormal findings may lead to stress testing, invasive coronary angiography, and potentially unnecessary revascularization without evidence that such a CAC-initiated diagnostic cascade improves clinical outcomes [24]. Accordingly, CAC should not be considered a routine screening strategy or a substitute for clinical cardiovascular risk assessment in PWH. This distinction has become particularly relevant following the 2026 ACC/AHA dyslipidemia guideline, which recommends statin therapy for primary prevention in PWH aged 40–75 years receiving stable ART regardless of LDL-C level [25]. Within this age group, CAC is therefore not required to establish statin eligibility; rather, selective imaging may be considered when additional information on atherosclerotic burden could meaningfully refine risk assessment or the intensity of preventive therapy, while recognizing the lack of HIV-specific randomized outcome data.

### Evidence for Statin Therapy

Statins remain the cornerstone of cardiovascular prevention and exert both lipid-lowering and pleiotropic anti-inflammatory effects, particularly relevant in the context of chronic immune activation observed in PWH.

Before REPRIEVE, evidence supporting statin therapy in PWH came mainly from observational cohorts and randomized trials using surrogate cardiovascular endpoints. Cohort data primarily suggested lower all-cause mortality with lipid-lowering therapy, whereas lower ASCVD event rates were generally not demonstrated; a 2021 US Veterans analysis was a notable exception, associating consistent lipid-lowering therapy with both lower mortality and lower ASCVD risk [26]. The inconsistency in ASCVD findings is more plausibly explained by confounding by indication, treatment selection, and healthy-user effects than by inadequate statistical power. Randomized trials showed favorable effects on subclinical atherosclerosis and inflammatory pathways [27, 28] but did not establish clinical cardiovascular event reduction. REPRIEVE therefore provided the first large randomized evidence of MACE reduction with statin therapy in PWH [10].

The REPRIEVE trial represents a landmark advance in this field. The trial enrolled 7,769 PWH aged 40–75 years who were receiving stable ART, had no known ASCVD, and had low-to-moderate estimated cardiovascular risk according to the 2013 Pooled Cohort Equations [10]. The median age was 50 years (interquartile range [IQR], 45–55), and the median estimated 10-year ASCVD risk was 4.5% (IQR, 2.1–7.0), underscoring that REPRIEVE primarily addressed cardiovascular prevention in a relatively young, low-risk population [10]. Over a median follow-up of 5.1 years, pitavastatin 4 mg daily reduced the incidence of MACE from 7.32 to 4.81 events per 1,000 person-years (hazard ratio, 0.65; 95% CI, 0.48–0.90), providing the first robust randomized evidence of cardiovascular event reduction with statin therapy in PWH at low-to-moderate predicted risk [10].

REPRIEVE provides direct randomized evidence for PWH aged 40–75 years. For PWH younger than 40 years, the absence of trial data should be interpreted alongside cohort evidence suggesting a numerically greater HIV-attributable relative risk of myocardial infarction at younger ages, although absolute event rates remain low and age-specific estimates are imprecise [1]. REPRIEVE therefore does not establish HIV status alone as an indication for lifelong statin therapy in otherwise low-risk younger adults, but it reinforces the importance of lifetime cardiovascular risk, conventional indications, and relevant HIV-related risk enhancers.

Baseline LDL-C levels in REPRIEVE were relatively modest, suggesting that benefit may not be fully explained by lipid lowering alone. Mechanistic studies in PWH show that statins can reduce immune activation and modify coronary plaque characteristics [28, 29]. More recent proteomic and transcriptomic data from REPRIEVE further implicated collagen remodeling and plaque stabilization, including an increase in procollagen C-endopeptidase enhancer 1 (PCOLCE) with pitavastatin [30].

Taken together, these data support a paradigm shift from a purely lipid-centered approach to a broader, risk-based and pathophysiology-driven strategy for cardiovascular prevention in PWH.

While these findings provide strong support for statin therapy in PWH, certain limitations should be acknowledged, including the relatively low baseline cardiovascular risk of the study population and the need for longer-term follow-up to fully assess durability of benefit.

### Clinical Interpretation of REPRIEVE

The REPRIEVE trial provides robust evidence supporting statin therapy for primary cardiovascular prevention in PWH aged 40–75 years at low-to-moderate estimated cardiovascular risk [10]. Its findings demonstrate that clinically meaningful cardiovascular protection can be achieved even in individuals who would not traditionally have been considered strong candidates for lipid-lowering therapy on the basis of conventional risk thresholds alone. However, the relative treatment effect should not be interpreted as implying an equivalent absolute clinical benefit across the entire spectrum of baseline cardiovascular risk.

REPRIEVE may be considered alongside JUPITER, an earlier primary-prevention trial that also demonstrated a substantial relative reduction in cardiovascular events among individuals who would not traditionally have been selected for statin therapy on the basis of LDL-C concentration alone. JUPITER randomized 17,802 apparently healthy adults without known cardiovascular disease, with LDL-C < 130 mg/dL and high-sensitivity C-reactive protein (hsCRP) ≥ 2 mg/L, to rosuvastatin 20 mg daily or placebo; the median age was 66 years, and rosuvastatin reduced the primary cardiovascular endpoint by 44% over a median follow-up of 1.9 years (hazard ratio, 0.56; 95% CI, 0.46–0.69) [31]. Both JUPITER and REPRIEVE therefore demonstrated cardiovascular benefit in populations with relatively modest baseline LDL-C levels and a relevant inflammatory context [10, 31]. However, the comparison should remain conceptual: elevated hsCRP was an explicit eligibility criterion in JUPITER, whereas REPRIEVE enrolled PWH according to age, ART status, and predicted cardiovascular risk without requiring elevated inflammatory biomarkers. Moreover, because statins lower both LDL-C and inflammatory biomarkers, neither trial can determine the extent to which event reduction was mediated by lipid lowering, anti-inflammatory effects, or their combination. REPRIEVE should therefore not be interpreted as proving that inflammation alone accounted for the observed cardiovascular benefit.

Importantly, the relative reduction in cardiovascular events observed in REPRIEVE should be interpreted alongside the absolute magnitude of benefit. The estimated number needed to treat over 5 years (NNT₅) to prevent one MACE varied substantially according to baseline 10-year ASCVD risk: 199 among participants with an estimated risk < 2.5%, 149 among those with a risk of 2.5% to < 5%, 53 among those with a risk of 5% to 10%, and 35 among those with a risk > 10% [10]. Thus, although the relative treatment effect was generally consistent across risk subgroups, the absolute benefit was considerably greater among participants with higher baseline cardiovascular risk. These estimates should be interpreted with some caution because the number of events within individual risk strata was limited. Nevertheless, this gradient in absolute benefit is clinically relevant and should inform shared decision-making, particularly for individuals at very low predicted risk, in whom the expected benefit of long-term preventive therapy should be considered alongside treatment burden, potential adverse effects, drug–drug interactions, and individual preferences [10].

REPRIEVE’s global recruitment and substantial representation of women and non-White participants strengthen generalizability within the population studied. However, PWH younger than 40 years and older than 75 years were not enrolled, so direct HIV-specific randomized evidence is absent outside this range. This should not be interpreted as evidence against statin therapy. At younger ages, cohort data suggest a potentially greater HIV-attributable relative risk despite low absolute event rates [1]. In adults older than 75 years, randomized general-population evidence indicates that reductions in major vascular events persist into older age, although primary-prevention evidence is less extensive [32]. In primary-prevention trials enrolling older adults, statins did not increase muscle-related symptoms, serious adverse events, or treatment discontinuation compared with placebo [33]. Chronological age alone should therefore not be used to withhold therapy; absolute ASCVD risk, life expectancy, frailty, comorbidity, polypharmacy, and individual preferences remain central.

From a clinical perspective, REPRIEVE establishes statin therapy as an evidence-based component of primary cardiovascular prevention in PWH aged 40–75 years, while the marked variation in absolute benefit across baseline risk strata underscores the continuing importance of individualized risk communication and shared decision-making [10].

### Who Should Receive Statins

The 2026 ACC/AHA multisociety dyslipidemia guideline recommends statin therapy for primary prevention in PWH aged 40–75 years receiving stable combination ART, irrespective of baseline LDL-C concentration (Class I, Level of Evidence B-R) [25]. This recommendation is principally informed by REPRIEVE, which demonstrated a significant reduction in first MACE with pitavastatin in PWH within this age range and at low-to-moderate estimated cardiovascular risk [10]. Accordingly, for PWH aged 40–75 years, statin therapy should no longer be restricted to those who cross conventional high-risk thresholds.

Guideline-level eligibility should not, however, be interpreted as implying uniform absolute benefit or eliminating the need for an informed treatment discussion. As demonstrated in REPRIEVE, the absolute reduction in cardiovascular events was substantially greater at higher baseline ASCVD risk, with an estimated 5-year NNT ranging from 199 in the lowest-risk stratum to 35 among participants with an estimated 10-year risk > 10% [10]. Quantitative risk assessment therefore remains clinically relevant for communicating the magnitude of expected benefit and for identifying individuals who may warrant more intensive LDL-C lowering [25]. Particularly among PWH at very low predicted risk, shared decision-making should address expected absolute benefit, long-term pill burden, potential muscle-related or metabolic adverse effects, drug–drug interactions, and individual preferences.

For PWH younger than 40 years, REPRIEVE provides no direct randomized evidence supporting routine statin initiation solely on the basis of HIV status [10]. However, cohort data suggest a numerically greater relative excess of myocardial infarction at younger ages while absolute event rates remain low [1]. Decisions should therefore follow general primary-prevention principles while incorporating LDL-C concentration, major conventional risk factors, lifetime cardiovascular risk, and relevant HIV-related risk enhancers. Lifestyle interventions remain central, while statin therapy may be appropriate when established general-population indications or sufficiently high lifetime risk are present [25].

For PWH older than 75 years, absence of direct REPRIEVE data should not itself be considered a reason to withhold statin therapy. General-population randomized evidence indicates that statin-associated reductions in major vascular events persist into older age, although primary-prevention evidence beyond 75 years is less extensive [32]. In primary-prevention trials enrolling older adults, statins did not increase common muscle-related symptoms or treatment discontinuation compared with placebo [33]. Consistent with the 2026 ACC/AHA guideline, moderate-intensity statin therapy may be reasonable in adults older than 75 years with an estimated life expectancy of at least 2.5 years after clinician–patient discussion of benefits and risks [25]. Decisions should still account for absolute cardiovascular risk, frailty, comorbidity, polypharmacy, drug–drug interactions, life expectancy, and treatment goals. CAC or other imaging is not required to establish statin eligibility in PWH aged 40–75 years and should remain selective [23–25].

### Choice of Statin and Prescribing Considerations

Statin selection in PWH should balance expected LDL cholesterol reduction, pleiotropic effects, long-term tolerability, cost, and the potential for drug–drug interactions with antiretroviral therapy, which remain central considerations in clinical practice (Table 1).

**Table 1:** Statin–antiretroviral therapy interactions and prescribing considerations

ART class or regimen	Generally compatible options	Use with caution	Avoid	Dose considerations	Key considerations
NRTI	All statins	None	None	No adjustment	No clinically relevant interactions
Unboosted INSTI (BIC, CAB, DTG, RAL)	Pitavastatin, atorvastatin, rosuvastatin, pravastatin	None routinely required	None specifically	No routine adjustment; verify the complete regimen	Low interaction potential
EVG/c-containing regimen	Pitavastatin or other statins according to interaction guidance	Atorvastatin, rosuvastatin	Simvastatin, lovastatin	Agent-specific dose limits and monitoring required	Manage as a cobicistat-boosted regimen
NNRTI	Atorvastatin, rosuvastatin	Simvastatin, lovastatin	None	Adjust as needed	Possible reduced statin levels (e.g., efavirenz)
Ritonavir- or cobicistat-boosted PI	Pitavastatin; atorvastatin, rosuvastatin, or pravastatin when compatible with the specific regimen	Atorvastatin, rosuvastatin, pravastatin	Simvastatin, lovastatin	Apply agent-specific dose limits; consult current interaction guidance	Interaction magnitude varies according to the PI, booster, and statin

Dose recommendations vary according to the specific statin, antiretroviral agent, and pharmacokinetic booster. Current prescribing information and dedicated HIV drug–interaction resources should be consulted before treatment initiation or dose escalation. Simvastatin and lovastatin are contraindicated with ritonavir- or cobicistat-boosted regimens. EVG/c should be considered a boosted regimen rather than grouped with unboosted integrase inhibitors

Abbreviations: *ART*, antiretroviral therapy; *BIC*, bictegravir; *CAB*, cabotegravir; *DTG*, dolutegravir; *EVG/c*, elvitegravir/cobicistat; *INSTI*, integrase strand transfer inhibitor; *NNRTI*, non-nucleoside reverse transcriptase inhibitor; *NRTI*, nucleos(t)ide reverse transcriptase inhibitor; *PI*, protease inhibitor; *RAL*, raltegravir

In terms of lipid-lowering efficacy, atorvastatin and rosuvastatin are among the most potent statins and are often required to achieve substantial LDL cholesterol reduction, particularly in individuals at higher cardiovascular risk [34–36]. Pitavastatin provides moderate-intensity LDL cholesterol lowering and is supported by the strongest HIV-specific outcome evidence, as it was the statin evaluated in the REPRIEVE trial [10]. Pravastatin has a favorable interaction profile but generally exhibits more modest lipid-lowering efficacy, which may limit its role when greater LDL cholesterol reduction is required [36].

Beyond LDL-C lowering, statins may exert plaque-modifying and immunomodulatory effects [37]. In SATURN-HIV, rosuvastatin reduced markers of monocyte, lymphocyte, and vascular activation [28]. The REPRIEVE mechanistic substudy subsequently showed that pitavastatin reduced noncalcified coronary plaque volume, plaque progression, and selected markers of lipid oxidation and arterial inflammation [29]. A later proteomic and transcriptomic analysis identified a potential collagen-mediated stabilization pathway: pitavastatin increased PCOLCE, a rate-limiting enzyme involved in collagen deposition, and greater PCOLCE increases were associated with reductions in noncalcified and fibro-fatty plaque independently of LDL-C changes. Collagen-related gene pathways were also enriched. PCOLCE was directionally associated with greater calcified plaque volume, although this was not statistically significant, a pattern consistent with—but not proving—a stabilization phenotype involving fibrosis and calcification [30]. These data suggest that collagen-mediated plaque stabilization may contribute to pitavastatin’s cardiovascular benefit alongside LDL-C lowering and other pleiotropic effects.

Drug–drug interactions are a major determinant of statin selection in PWH and should be evaluated according to the specific statin–antiretroviral combination rather than the antiretroviral class alone. Ritonavir and cobicistat inhibit cytochrome P450 enzymes and drug transporters and may substantially increase exposure to selected statins, whereas some non-nucleoside reverse transcriptase inhibitors may reduce statin exposure through enzyme induction. Unboosted integrase strand transfer inhibitors and nucleoside reverse transcriptase inhibitors generally have limited potential for clinically relevant interactions; however, elvitegravir co-formulated with cobicistat should be managed as a pharmacokinetically boosted regimen [18, 36].

Pitavastatin has minimal cytochrome P450 metabolism and a generally favorable interaction profile, but it should not be regarded as universally preferred for all boosted or otherwise complex antiretroviral regimens. Similarly, although pravastatin has limited CYP3A4 metabolism, its comparatively modest LDL-C–lowering efficacy and the possibility of transporter-mediated interactions should be considered. Atorvastatin and rosuvastatin remain appropriate options when greater LDL-C reduction is required, provided that agent-specific dose limitations and monitoring recommendations are followed for the antiretroviral regimen being used [18, 36]. Simvastatin and lovastatin remain contraindicated with ritonavir- or cobicistat-boosted regimens because of the risk of markedly increased exposure and severe muscle toxicity [18, 36]. Overall, statin selection should balance the required LDL-C reduction, the exact antiretroviral regimen, interaction potential, tolerability, and individual clinical characteristics rather than relying on a single preferred statin for all PWH receiving boosted therapy.

Long-term tolerability and safety should also guide treatment choice. Pitavastatin showed a favorable safety profile in REPRIEVE, although muscle-related symptoms and incident diabetes were slightly more frequent than with placebo [10]. Pravastatin has low interaction potential but more modest LDL-C–lowering efficacy, whereas atorvastatin and rosuvastatin provide greater lipid lowering and require closer monitoring when clinically relevant interactions or other adverse-effect risk factors are present. In primary-prevention trials enrolling older adults, statins did not increase muscle-related symptoms, serious adverse events, or treatment discontinuation compared with placebo [33]. Monitoring should therefore be guided by symptoms, comorbidities, frailty, renal or hepatic impairment, polypharmacy, and interacting therapies rather than chronological age alone [18, 34–36].

Cost and access are important considerations in real-world practice. Generic atorvastatin and rosuvastatin are widely available and relatively inexpensive in many settings, whereas pitavastatin may be less accessible or more costly despite its favorable interaction profile and HIV-specific outcome evidence [36].

Overall, the choice of statin in PWH should be individualized according to the required magnitude of LDL-C reduction, cardiovascular risk, the specific antiretroviral regimen and its interaction profile, previous tolerability, comorbidities, cost, and access.

### Non-Statin Lipid-Lowering Therapies

Evidence supporting non-statin lipid-lowering therapies in PWH remains substantially less robust than that available for statins. Although ezetimibe, PCSK9 inhibitors, and bempedoic acid have demonstrated cardiovascular benefit in large randomized trials conducted in the general population, most of these data derive from populations with established ASCVD or otherwise high cardiovascular risk, and the absolute reductions in cardiovascular events have generally been modest [38–41]. Importantly, no adequately powered randomized trial has yet demonstrated that any non-statin lipid-lowering therapy reduces cardiovascular events specifically in PWH. Therefore, cardiovascular outcome data from the general population should be extrapolated to PWH with appropriate caution. The principal outcome trials and their absolute treatment effects are summarized in Supplementary Table S1.

Ezetimibe is a well-established adjunct to statin therapy because of its modest additional LDL-C reduction, favorable tolerability, and limited potential for clinically relevant drug–drug interactions. In IMPROVE-IT, conducted in participants with recent acute coronary syndrome receiving simvastatin, the addition of ezetimibe reduced the 7-year Kaplan–Meier rate of the primary cardiovascular endpoint from 34.7% to 32.7%, corresponding to an absolute difference of 2.0% points [38]. Small studies in PWH have demonstrated improvements in lipid parameters with ezetimibe, including when added to rosuvastatin, but these studies were short, involved relatively few participants, and were not designed to assess cardiovascular events [42]. Thus, its cardiovascular benefit in PWH is inferred primarily from evidence generated outside the HIV population.

PCSK9 monoclonal antibodies provide substantially greater LDL-C lowering and have demonstrated cardiovascular outcome benefits in high-risk populations without specific selection for HIV. In FOURIER, evolocumab reduced the primary cardiovascular endpoint from 11.3% to 9.8% over a median follow-up of 2.2 years, whereas in ODYSSEY OUTCOMES, alirocumab reduced the corresponding primary endpoint from 11.1% to 9.5% over a median follow-up of 2.8 years [39, 40]. HIV-specific evidence is available for evolocumab from the randomized BEIJERINCK trial, in which treatment produced a placebo-corrected 56.9% reduction in LDL-C at 24 weeks and was generally well tolerated [43]. However, BEIJERINCK was a lipid-efficacy and safety trial rather than a cardiovascular outcome trial, and whether PCSK9 inhibition produces a comparable reduction in cardiovascular events specifically in PWH remains unproven.

Bempedoic acid represents an additional oral option, particularly for individuals who cannot tolerate adequate statin therapy. In CLEAR Outcomes, which enrolled statin-intolerant individuals with established cardiovascular disease or at high cardiovascular risk, the primary cardiovascular endpoint occurred in 11.7% of participants receiving bempedoic acid and 13.3% receiving placebo over a median follow-up of 40.6 months (hazard ratio, 0.87; 95% CI, 0.79–0.96) [41]. Dedicated efficacy and cardiovascular outcome data in PWH are currently lacking. Accordingly, the choice to add a non-statin agent in PWH should be individualized according to residual cardiovascular risk, achieved LDL-C level, statin tolerance, treatment burden, potential drug–drug interactions, cost and access, while recognizing that HIV-specific cardiovascular outcome evidence remains limited.

### ART and Lipid Profile: the Role of Tenofovir Disoproxil Fumarate

Antiretroviral regimen composition can influence lipid metabolism. TDF-containing regimens have generally been associated with more favorable lipid profiles than TAF-containing regimens, and observational switch studies have reported increases in LDL-C and triglycerides after TDF was replaced by TAF. In the OPERA cohort, 6,451 PWH who switched from TDF to TAF experienced early increases in LDL-C and triglycerides, with similar patterns among those who maintained the other components of their antiretroviral regimen [44]. However, these findings concern surrogate metabolic outcomes and do not demonstrate that retaining or introducing TDF reduces cardiovascular events.

The randomized ACTG A5391 (DO-IT) trial provides important context. This 48-week, open-label, three-group trial randomized 147 PWH with obesity and virologic suppression receiving bictegravir, dolutegravir, or raltegravir with TAF/FTC to switch to DOR plus TAF/FTC, switch to DOR plus TDF/FTC, or continue INSTI plus TAF/FTC [45]. At week 48, switching did not produce clinically meaningful between-group differences in weight change or metabolic health, and no treatment differences were observed in fasting lipids, insulin resistance, fat mass, or bone mineral density [45]. These findings do not support switching a virologically suppressive INSTI plus TAF regimen to a doravirine-based regimen solely to obtain weight or metabolic benefit.

From a clinical perspective, a potentially more favorable lipid profile with TDF should be regarded as a secondary consideration rather than as a lipid-lowering strategy. Any possible metabolic advantage must be balanced against renal and bone safety, comorbidities, prior toxicities, virologic and resistance history, HBV status, regimen simplicity, and individual preferences [36, 46]. ART modification should therefore not be used as a substitute for evidence-based lipid-lowering therapy. When a regimen change is otherwise clinically appropriate, the expected metabolic effects of TDF and TAF may be incorporated into individualized decision-making.

### Monitoring and Follow-up

PWH initiating statin therapy should be monitored for lipid response, treatment adherence, and potential adverse effects. A structured follow-up strategy is essential to optimize treatment effectiveness and ensure long-term adherence.

Initial lipid reassessment is typically recommended within 4–12 weeks after statin initiation, followed by periodic monitoring to guide dose adjustment and evaluate therapeutic response [34, 35]. In addition to lipid levels, clinicians should assess adherence, tolerability, and potential drug–drug interactions with antiretroviral therapy [18, 36].

Routine monitoring of liver enzymes or creatine kinase (CK) is not required in asymptomatic individuals but should be considered in the presence of clinical symptoms suggestive of hepatotoxicity or myopathy [34, 35]. Particular attention should be given to individuals with comorbidities, polypharmacy, or those receiving boosted antiretroviral regimens, in whom the risk of adverse effects may be increased.

Periodic reassessment of overall cardiovascular risk is recommended, taking into account changes in clinical status, antiretroviral therapy, and the cumulative burden of HIV-related risk factors over time [3, 36]. This dynamic approach is particularly important in PWH, in whom cardiovascular risk may evolve independently of traditional risk scores.

**Fig. 1:**
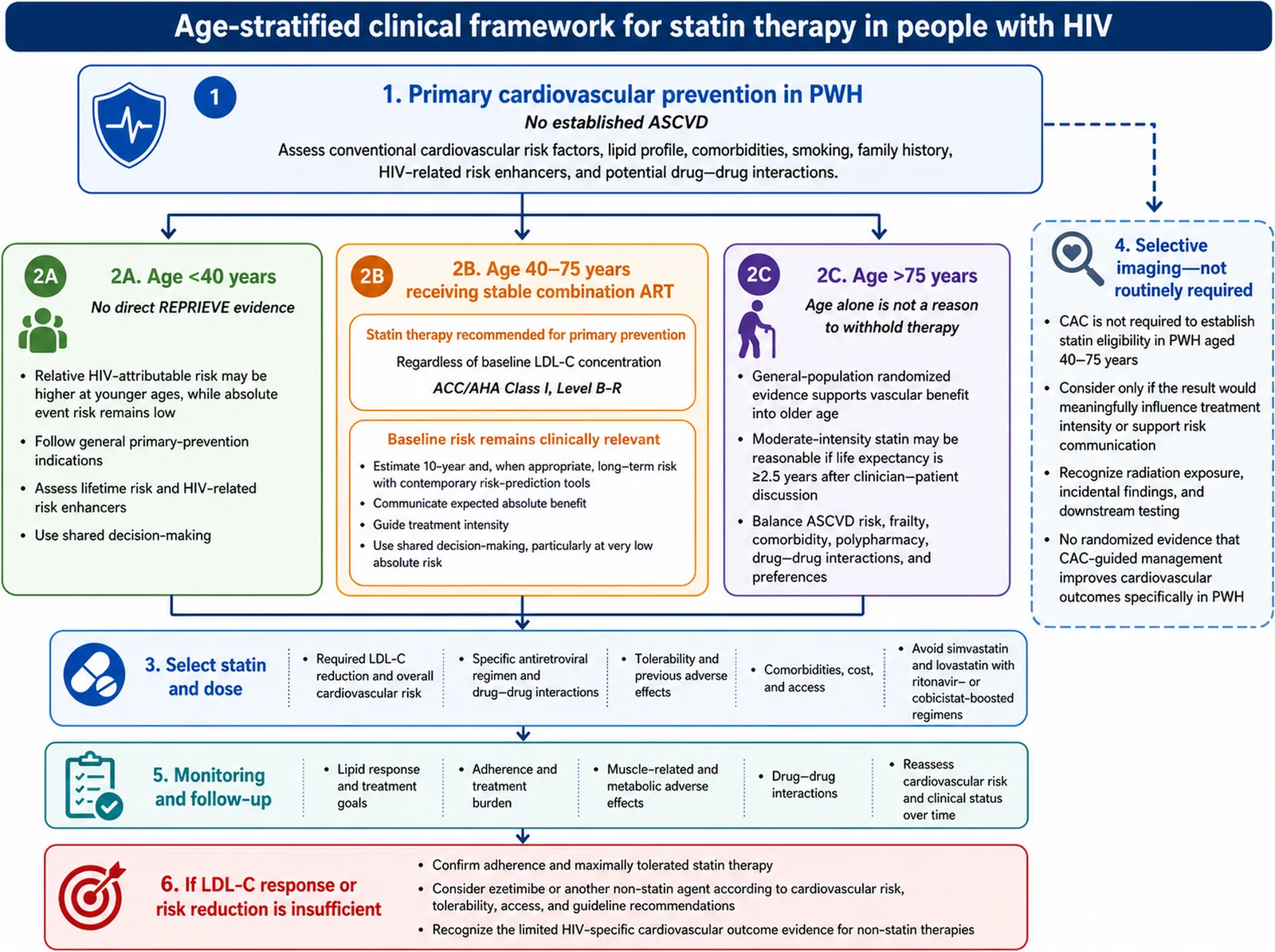
Age-stratified clinical framework for statin therapy in people with HIV. The approach to statin therapy for primary cardiovascular prevention in people with HIV (PWH) should account for both the age range directly studied in REPRIEVE and evidence available outside that range. In PWH aged 40–75 years receiving stable combination antiretroviral therapy (ART), the 2026 ACC/AHA multisociety dyslipidemia guideline recommends statin therapy to reduce the risk of a first atherosclerotic cardiovascular disease (ASCVD) event. Baseline cardiovascular risk assessment remains useful for communicating expected absolute benefit and informing treatment intensity because the absolute benefit observed in REPRIEVE varied across risk strata. For PWH younger than 40 years, REPRIEVE provides no direct randomized evidence; however, cohort data suggest that HIV-attributable relative cardiovascular risk may be particularly relevant at younger ages despite low absolute event rates, so decisions should integrate general primary-prevention indications, lifetime risk, and HIV-related risk enhancers. For PWH older than 75 years, absence of REPRIEVE data should not itself prompt withholding statin therapy. General-population randomized evidence supports vascular benefit into older age, and current guidelines consider initiation of moderate-intensity statin therapy reasonable in selected adults older than 75 years with adequate life expectancy after clinician–patient discussion. Decisions should still account for frailty, comorbidity, polypharmacy, drug–drug interactions, life expectancy, and individual goals and preferences. Coronary artery calcium (CAC) scoring is not required to establish statin eligibility in PWH aged 40–75 years and should be considered selectively only when the result could meaningfully influence treatment intensity or support risk communication. Statin selection and dosing should account for the required LDL-C reduction, antiretroviral regimen, potential drug–drug interactions, tolerability, comorbidities, cost, and access. Follow-up should assess lipid response, adherence, adverse effects, drug–drug interactions, and the need for treatment intensification [1, 10, 23–25, 32, 33]. Abbreviations: ART, antiretroviral therapy; ASCVD, atherosclerotic cardiovascular disease; CAC, coronary artery calcium; CKD, chronic kidney disease; LDL-C, low-density lipoprotein cholesterol; PWH, people with HIV

Figure 1 summarizes an age-stratified clinical framework for statin therapy in PWH, integrating the population directly supported by REPRIEVE, current guideline recommendations, individualized risk communication, statin selection, the selective use of cardiovascular imaging, and longitudinal follow-up.

### Future Perspectives

The REPRIEVE trial and the 2026 ACC/AHA multisociety dyslipidemia guideline have substantially changed primary cardiovascular prevention in PWH. For those aged 40–75 years receiving stable combination ART, the central questions now concern expected absolute benefit, treatment intensity, long-term tolerability, and individual preferences rather than basic statin eligibility [10, 25]. HIV-specific evidence gaps remain outside the REPRIEVE age range. In younger PWH, cohort data suggest that relative HIV-attributable cardiovascular risk may be heightened despite low short-term absolute risk [1]. In adults older than 75 years, general-population randomized evidence and current guidelines support considering statin therapy rather than withholding it solely because of age, while dedicated HIV-specific outcome data remain lacking [25, 32, 33].

Improved cardiovascular risk stratification remains an important research priority. Contemporary tools such as the PREVENT equations may provide more current estimates of 10-year and 30-year cardiovascular risk, but they were not developed specifically for PWH and do not directly incorporate HIV-related variables [9, 25]. Future studies should determine whether incorporating factors such as cumulative exposure to viremia, nadir CD4 + T-cell count, selected antiretroviral exposures, and markers of persistent inflammation meaningfully improves prediction beyond conventional models. Similarly, the incremental value of CAC scoring or other imaging modalities should be evaluated prospectively; current HIV-specific evidence remains predominantly observational, and no randomized study has demonstrated that imaging-guided management improves cardiovascular outcomes in PWH [23, 24].

Lp(a) represents another potential component of risk refinement. Current guidelines recommend measuring Lp(a) at least once in adulthood and consider concentrations ≥ 125 nmol/L or ≥ 50 mg/dL to be elevated [25]. However, HIV-specific evidence remains limited and inconsistent. A small cross-sectional study reported higher Lp(a) concentrations and an inverse association with coronary endothelial function in PWH, whereas the larger COCOMO analysis found no independent association between HIV status and elevated Lp(a) [47, 48]. Prospective studies are needed to determine whether Lp(a) provides incremental prognostic information or identifies PWH who derive greater benefit from intensified preventive strategies.

Additional priorities include establishing the long-term cardiovascular effectiveness and safety of non-statin lipid-lowering therapies specifically in PWH and clarifying whether inflammation-targeted interventions can provide benefit beyond established risk-factor management [49]. Effective implementation will also require improved cardiovascular risk communication, systematic assessment of drug–drug interactions, support for long-term adherence, and integration of prevention into the broader management of ageing, multimorbidity, frailty, and polypharmacy. Future strategies should therefore combine evidence-based lipid lowering with individualized, longitudinal, and person-centred cardiovascular care.

## Conclusion

REPRIEVE established statin therapy as an evidence-based component of primary cardiovascular prevention in PWH aged 40–75 years receiving ART and at low-to-moderate estimated cardiovascular risk [10]. Consistent with these findings, the 2026 ACC/AHA multisociety dyslipidemia guideline recommends statin therapy for primary prevention in PWH aged 40–75 years receiving stable combination ART, irrespective of baseline LDL-C concentration [25]. This represents an important change in clinical practice, extending preventive therapy beyond conventional high-risk thresholds within the population directly supported by randomized evidence.

Guideline-level eligibility does not imply uniform absolute benefit. Baseline cardiovascular risk remains important for communicating expected benefit, informing treatment intensity, and supporting shared decision-making. Decisions should also account for antiretroviral drug–drug interactions, tolerability, treatment burden, comorbidities, and individual preferences. Outside the REPRIEVE age range, lack of direct HIV-specific randomized evidence should not be equated with lack of potential benefit. Among PWH younger than 40 years, absolute event rates are low but cohort data suggest a potentially greater HIV-attributable relative risk, supporting attention to lifetime risk and HIV-related risk enhancers [1]. Among adults older than 75 years, general-population randomized evidence supports vascular benefit from statins, while trials in older primary-prevention populations show no excess of common muscle symptoms or treatment discontinuation compared with placebo; current guidelines therefore consider moderate-intensity statin therapy reasonable in selected older adults with adequate life expectancy after clinician–patient discussion [25, 32, 33]. Cardiovascular imaging should remain selective, and further studies are needed to improve HIV-specific risk prediction and define cardiovascular outcome benefits of non-statin therapies in PWH.

## Supplementary Information

Below is the link to the electronic supplementary material.

Supplementary Material 1 (DOCX 51.9 KB)

## Data Availability

No datasets were generated or analysed during the current study.

## References

[1] Freiberg MS, Chang CC, Kuller LH, et al. HIV infection and the risk of acute myocardial infarction. JAMA Intern Med. 2013;173(8):614–22. 10.1001/jamainternmed.2013.3728. 23459863 PMC4766798

[2] Triant VA. Cardiovascular disease and HIV infection. Curr HIV/AIDS Rep. 2013;10(3):199–206. 10.1007/s11904-013-0168-6. 23793823 PMC3964878

[3] Feinstein MJ, Hsue PY, Benjamin LA, et al. Characteristics, Prevention, and Management of Cardiovascular Disease in People Living With HIV: A Scientific Statement From the American Heart Association. Circulation. 2019;140(2):e98–124. 10.1161/CIR.0000000000000695. 31154814 PMC7993364

[4] Hsue PY, Hunt PW, Schnell A, et al. Role of viral replication, antiretroviral therapy, and immunodeficiency in HIV-associated atherosclerosis. AIDS. 2009;23(9):1059–67. 10.1097/QAD.0b013e32832b514b. 19390417 PMC2691772

[5] Kuller LH, Tracy R, Belloso W, INSIGHT SMART Study Group, et al. Inflammatory and coagulation biomarkers and mortality in patients with HIV infection. PLoS Med. 2008;5(10):e203. 10.1371/journal.pmed.0050203. 18942885 PMC2570418

[6] Duprez DA, Neuhaus J, Kuller LH, INSIGHT SMART Study Group, et al. Inflammation, coagulation and cardiovascular disease in HIV-infected individuals. PLoS ONE. 2012;7(9):e44454. 10.1371/journal.pone.0044454. 22970224 PMC3438173

[7] Triant VA, Lee H, Hadigan C, Grinspoon SK. Increased acute myocardial infarction rates and cardiovascular risk factors among patients with human immunodeficiency virus disease. J Clin Endocrinol Metab. 2007;92(7):2506–12. 10.1210/jc.2006-2190. 17456578 PMC2763385

[8] Crane HM, Paramsothy P, Drozd DR, et al. Types of Myocardial Infarction Among Human Immunodeficiency Virus-Infected Individuals in the United States. JAMA Cardiol. 2017;2(3):260–7. 10.1001/jamacardio.2016.5139. 28052152 PMC5538773

[9] Achhra AC, Lyass A, Borowsky L, et al. Assessing Cardiovascular Risk in People Living with HIV: Current Tools and Limitations. Curr HIV/AIDS Rep. 2021;18(4):271–9. 10.1007/s11904-021-00567-w. 34247329 PMC8733948

[10] Grinspoon SK, Fitch KV, Zanni MV, et al. REPRIEVE Trial Investigators. Pitavastatin to Prevent Cardiovascular Disease in HIV Infection. N Engl J Med. 2023;389(8):687–99. 10.1056/NEJMoa2304146. 37486775 PMC10564556

[11] Mdodo R, Frazier EL, Dube SR, et al. Cigarette smoking prevalence among adults with HIV compared with the general adult population in the United States: cross-sectional surveys. Ann Intern Med. 2015;162(5):335–44. 10.7326/M14-0954. 25732274

[12] Sandler NG, Wand H, Roque A, INSIGHT SMART Study Group, et al. Plasma levels of soluble CD14 independently predict mortality in HIV infection. J Infect Dis. 2011;203(6):780–90. 10.1093/infdis/jiq118. 21252259 PMC3071127

[13] Lo J, Abbara S, Shturman L, et al. Increased prevalence of subclinical coronary atherosclerosis detected by coronary computed tomography angiography in HIV-infected men. AIDS. 2010;24(2):243–53. 10.1097/QAD.0b013e328333ea9e. 19996940 PMC3154841

[14] D:A:D Study Group, Sabin CA, Worm SW, Weber R, et al. Use of nucleoside reverse transcriptase inhibitors and risk of myocardial infarction in HIV-infected patients enrolled in the D:A:D study: a multi-cohort collaboration. Lancet. 2008;371(9622):1417–26. 10.1016/S0140-6736(08)60423-7. 18387667 PMC2688660

[15] Davies Smith E, Malvestutto C, Ribaudo HJ, et al. Cardiovascular Hazards of Abacavir- Versus Tenofovir-Containing Antiretroviral Therapies: Insights From an Analysis of the REPRIEVE Trial Cohort. Open Forum Infect Dis. 2025;12(4):ofaf177. 10.1093/ofid/ofaf177. 40207047 PMC11979587

[16] Sax PE, Erlandson KM, Lake JE, et al. Weight Gain Following Initiation of Antiretroviral Therapy: Risk Factors in Randomized Comparative Clinical Trials. Clin Infect Dis. 2020;71(6):1379–89. 10.1093/cid/ciz999. 31606734 PMC7486849

[17] Ryom L, Lundgren JD, El-Sadr W, et al. D:A:D Study Group. Cardiovascular disease and use of contemporary protease inhibitors: the D:A:D international prospective multicohort study. Lancet HIV. 2018;5(6):e291–300. 10.1016/S2352-3018(18)30043-2. 29731407

[18] European AIDS, Clinical Society (EACS). EACS Guidelines, Version 13.0. October 2025. Available at: https://www.eacsociety.org/guidelines/eacs-guidelines/. Accessed 1 Aug 2026.

[19] Hsue PY, Lo JC, Franklin A, et al. Progression of atherosclerosis as assessed by carotid intima-media thickness in patients with HIV infection. Circulation. 2004;109(13):1603–8. 10.1161/01.CIR.0000124480.32233.8A. 15023877

[20] Hsue PY, Scherzer R, Hunt PW, et al. Carotid intima-media thickness progression in HIV-infected adults occurs preferentially at the carotid bifurcation and is predicted by inflammation. J Am Heart Assoc. 2012;1(2):e000422. 10.1161/JAHA.111.000422. PMC3487373 23130122

[21] Lorenz MW, Markus HS, Bots ML, Rosvall M, Sitzer M. Prediction of clinical cardiovascular events with carotid intima-media thickness: a systematic review and meta-analysis. Circulation. 2007;115(4):459–67. 10.1161/CIRCULATIONAHA.106.628875. 17242284

[22] Pereira B, Mazzitelli M, Milinkovic A, et al. Use of Coronary Artery Calcium Scoring to Improve Cardiovascular Risk Stratification and Guide Decisions to Start Statin Therapy in People Living With HIV. J Acquir Immune Defic Syndr. 2020;85(1):98–105. 10.1097/QAI.0000000000002400. 32398558

[23] Soares C, Samara A, Yuyun MF, et al. Coronary Artery Calcification and Plaque Characteristics in People Living With HIV: A Systematic Review and Meta-Analysis. J Am Heart Assoc. 2021;10(19):e019291. 10.1161/JAHA.120.019291. 34585590 PMC8649136

[24] Mandrola J, Foy A. The Case Against Coronary Artery Calcium Scoring for Cardiovascular Disease Risk Assessment. Am Fam Physician. 2019;100(12):734–5. 31845777

[25] Blumenthal RS, Morris PB, Gaudino M, et al. 2026 ACC/AHA/AACVPR/ABC/ACPM/ADA/AGS/APhA/ASPC/NLA/PCNA Guideline on the Management of Dyslipidemia: A Report of the American College of Cardiology/American Heart Association Joint Committee on Clinical Practice Guidelines. J Am Coll Cardiol. 2026;87(19):2624–757. 10.1016/j.jacc.2025.11.016. 41824590

[26] Drechsler H, Ayers C, Cutrell J, Arasaratnam R, Bedimo R. Consistent use of lipid lowering therapy in HIV infection is associated with low mortality. BMC Infect Dis. 2021;21(1):150. 10.1186/s12879-021-05787-4. 33546621 PMC7866454

[27] Longenecker CT, Sattar A, Gilkeson R, McComsey GA. Rosuvastatin slows progression of subclinical atherosclerosis in patients with treated HIV infection. AIDS. 2016;30(14):2195–203. 10.1097/QAD.0000000000001167. 27203715 PMC5007142

[28] Funderburg NT, Jiang Y, Debanne SM, et al. Rosuvastatin reduces vascular inflammation and T-cell and monocyte activation in HIV-infected subjects on antiretroviral therapy. J Acquir Immune Defic Syndr. 2015;68(4):396–404. 10.1097/QAI.0000000000000478. 25514794 PMC4334694

[29] Lu MT, Ribaudo H, Foldyna B, REPRIEVE Trial Writing Group, et al. Effects of Pitavastatin on Coronary Artery Disease and Inflammatory Biomarkers in HIV: Mechanistic Substudy of the REPRIEVE Randomized Clinical Trial. JAMA Cardiol. 2024;9(4):323–34. 10.1001/jamacardio.2023.5661. 38381407 PMC10882511

[30] Kolossváry M, Schnittman SR, Zanni MV, et al. Pitavastatin, Procollagen Pathways, and Plaque Stabilization in Patients With HIV: A Secondary Analysis of the REPRIEVE Randomized Clinical Trial. JAMA Cardiol. 2025;10(3):254–64. 10.1001/jamacardio.2024.4115. 39661372 PMC11771813

[31] Ridker PM, Danielson E, Fonseca FAH, JUPITER Study Group, et al. Rosuvastatin to Prevent Vascular Events in Men and Women with Elevated C-Reactive Protein. N Engl J Med. 2008;359(21):2195–207. 10.1056/NEJMoa0807646. 18997196

[32] Cholesterol Treatment Trialists’ Collaboration. Efficacy and safety of statin therapy in older people: a meta-analysis of individual participant data from 28 randomised controlled trials. Lancet. 2019;393(10170):407–15. 10.1016/S0140-6736(18)31942-1. 30712900 PMC6429627

[33] Zhou Z, Albarqouni L, Curtis AJ, Breslin M, Nelson M. The Safety and Tolerability of Statin Therapy in Primary Prevention in Older Adults: A Systematic Review and Meta-analysis. Drugs Aging. 2020;37(3):175–85. 10.1007/s40266-019-00736-y. 31919804

[34] Mach F, Baigent C, Catapano AL, ESC Scientific Document Group. 2019 ESC/EAS Guidelines for the management of dyslipidaemias: lipid modification to reduce cardiovascular risk. Eur Heart J. 2020;41(1):111–88. 10.1093/eurheartj/ehz455. 31504418

[35] Grundy SM, Stone NJ, Bailey AL, PCNA Guideline on the Management of Blood Cholesterol. A Report of the American College of Cardiology/American Heart Association Task Force on Clinical Practice Guidelines. J Am Coll Cardiol. 2019;73(24):e285–350.2018 AHA/ACC/AACVPR/AAPA. 10.1016/j.jacc.2018.11.003. /ABC/ACPM/ADA/AGS/APhA/ASPC/NLA. 2018 AHA/ACC/AACVPR/AAPA. 30423393

[36] Panel for the Use of Antiretroviral Agents in Adults and Adolescents With HIV. Guidelines for the Use of Antiretroviral Agents in Adults and Adolescents With HIV. Department of Health and Human Services. Updated May 27. 2026. Available at: https://clinicalinfo.hiv.gov/en/guidelines/hiv-clinical-guidelines-adult-and-adolescent-arv. Accessed August 1, 2026.

[37] Davignon J. Beneficial cardiovascular pleiotropic effects of statins. Circulation. 2004;109(23 Suppl 1):III39–43. 10.1161/01.CIR.0000131517.20177.5a. 15198965

[38] Cannon CP, Blazing MA, Giugliano RP, et al. Ezetimibe Added to Statin Therapy after Acute Coronary Syndromes. N Engl J Med. 2015;372(25):2387–97. 10.1056/NEJMoa1410489. 26039521

[39] Sabatine MS, Giugliano RP, Keech AC, et al. Evolocumab and Clinical Outcomes in Patients with Cardiovascular Disease. N Engl J Med. 2017;376(18):1713–22. 10.1056/NEJMoa1615664. 28304224

[40] Schwartz GG, Steg PG, Szarek M, et al. Alirocumab and Cardiovascular Outcomes after Acute Coronary Syndrome. N Engl J Med. 2018;379(22):2097–107. 10.1056/NEJMoa1801174. 30403574

[41] Nissen SE, Lincoff AM, Brennan D, et al. Bempedoic Acid and Cardiovascular Outcomes in Statin-Intolerant Patients. N Engl J Med. 2023;388(15):1353–64. 10.1056/NEJMoa2215024. 36876740

[42] Saeedi R, Johns K, Frohlich J, Bennett MT, Bondy G. Lipid lowering efficacy and safety of ezetimibe combined with rosuvastatin compared with titrating rosuvastatin monotherapy in HIV-positive patients. Lipids Health Dis. 2015;14:57. 10.1186/s12944-015-0054-x. 26087958 PMC4488121

[43] Boccara F, Kumar PN, Caramelli B, et al. Evolocumab in HIV-Infected Patients With Dyslipidemia: Primary Results of the Randomized, Double-Blind BEIJERINCK Study. J Am Coll Cardiol. 2020;75(20):2570–84. 10.1016/j.jacc.2020.03.025. 32234462

[44] Mallon PWG, Brunet L, Hsu RK, et al. Lipid Changes After Switch From TDF to TAF in the OPERA Cohort: LDL Cholesterol and Triglycerides. Open Forum Infect Dis. 2022;9(1):ofab621. 10.1093/ofid/ofab621. 35028335 PMC8753026

[45] Koethe JR, Lake JE, Kantor A, et al. A 48-Week, Randomized Controlled Trial of Doravirine for Individuals With HIV and Obesity on Integrase Inhibitors and Tenofovir Alafenamide: The Do IT Study (ACTG A5391). Clin Infect Dis. 2026;83(1):e81–9. 10.1093/cid/ciag196. 41847923 PMC13393121

[46] Gupta SK, Post FA, Arribas JR, et al. Renal safety of tenofovir alafenamide vs. tenofovir disoproxil fumarate: a pooled analysis of 26 clinical trials. AIDS. 2019;33(9):1455–65. 10.1097/QAD.0000000000002223. 30932951 PMC6635043

[47] Kikuchi DS, Kwapong YA, Schär M, et al. Lipoprotein(a) Is Elevated and Inversely Related to Coronary Endothelial Function in People With HIV. J Am Heart Assoc. 2024;13(23):e035975. 10.1161/JAHA.124.035975. 39575706 PMC11681595

[48] Stoltz TT, Jensen AMR, Roed AKH, et al. Plasma levels of lipoprotein(a) in persons with HIV compared to the general population. AIDS. 2026;40(1):35–42. 10.1097/QAD.0000000000004349. 40971414

[49] Obare LM, Temu T, Mallal SA, Wanjalla CN. Inflammation in HIV and Its Impact on Atherosclerotic Cardiovascular Disease. Circ Res. 2024;134(11):1515–45. 10.1161/CIRCRESAHA.124.323891. 38781301 PMC11122788

