# Supplementary material for: Primary Cardiovascular Prevention in People Living With HIV: Rethinking Statin Therapy in the Post-REPRIEVE Era
Source: Curr HIV/AIDS Rep. 2026 Sep 16;23(1):28. doi: 10.1007/s11904-026-00797-w (PMC13582062; doi:10.1007/s11904-026-00797-w)
Supplement: Supplementary file 1 [file 11904_2026_797_MOESM1_ESM.docx]

**Supplementary Table S1. Cardiovascular outcome evidence for major non-statin lipid-lowering therapies**

| **Agent / trial** | **Population and background therapy** | **Primary cardiovascular endpoint** | **Active treatment** | **Control** | **Absolute difference** | **Relative effect** | **HIV-specific outcome evidence** |
| --- | --- | --- | --- | --- | --- | --- | --- |
| Ezetimibe IMPROVE-IT [38] | 18,144 participants after acute coronary syndrome; simvastatin background therapy; 7-year Kaplan-Meier estimate | Cardiovascular death, major coronary event, or nonfatal stroke | 32.7% | 34.7% | 2.0 percentage points lower | HR 0.936 (95% CI, 0.89-0.99) | No cardiovascular outcome trial in PWH. Small, short-term studies demonstrated lipid efficacy only [42]. |
| Evolocumab FOURIER [39] | 27,564 participants with established ASCVD receiving statin therapy; median follow-up 2.2 years | Cardiovascular death, myocardial infarction, stroke, hospitalization for unstable angina, or coronary revascularization | 9.8% | 11.3% | 1.5 percentage points lower | HR 0.85 (95% CI, 0.79-0.92) | BEIJERINCK demonstrated LDL-C efficacy and safety with evolocumab, but was not a cardiovascular outcome trial [43]. |
| Alirocumab ODYSSEY OUTCOMES [40] | 18,924 participants after acute coronary syndrome receiving high-intensity or maximally tolerated statin therapy; median follow-up 2.8 years | Coronary heart disease death, nonfatal myocardial infarction, ischemic stroke, or hospitalization for unstable angina | 9.5% | 11.1% | 1.6 percentage points lower | HR 0.85 (95% CI, 0.78-0.93) | No dedicated cardiovascular outcome trial in PWH. |
| Bempedoic acid CLEAR Outcomes [41] | 13,970 statin-intolerant participants with established cardiovascular disease or at high cardiovascular risk; median follow-up 40.6 months | Cardiovascular death, nonfatal myocardial infarction, nonfatal stroke, or coronary revascularization | 11.7% | 13.3% | 1.6 percentage points lower | HR 0.87 (95% CI, 0.79-0.96) | No dedicated efficacy or cardiovascular outcome trial in PWH. |

Absolute differences are presented as percentage-point reductions in the trial-specific primary endpoint and should not be compared directly across trials because study populations, endpoint definitions, and follow-up durations differed.

No adequately powered randomized cardiovascular outcome trial of ezetimibe, PCSK9 inhibition, or bempedoic acid has been conducted specifically in PWH.

Abbreviations: ACS, acute coronary syndrome; ASCVD, atherosclerotic cardiovascular disease; CHD, coronary heart disease; CI, confidence interval; CV, cardiovascular; HR, hazard ratio; LDL-C, low-density lipoprotein cholesterol; MI, myocardial infarction; PCSK9, proprotein convertase subtilisin/kexin type 9; PWH, people with HIV.
